# Level-K Classification from EEG Signals Using Transfer Learning

**DOI:** 10.3390/s21237908

**Published:** 2021-11-27

**Authors:** Dor Mizrahi, Inon Zuckerman, Ilan Laufer

**Affiliations:** Department of Industrial Engineering and Management, Ariel University, Ariel 4076414, Israel; inonzu@ariel.ac.il (I.Z.); ilanl@ariel.ac.il (I.L.)

**Keywords:** level-k, EEG, classification, transfer learning, tacit coordination

## Abstract

Tacit coordination games are games in which communication between the players is not allowed or not possible. In these games, the more salient solutions, that are often perceived as more prominent, are referred to as *focal points.* The level-k model states that players’ decisions in tacit coordination games are a consequence of applying different decision rules at different depths of reasoning (level-k). A player at $L_{k=0}$ will randomly pick a solution, whereas a $L_{k\geq 1}$ player will apply their strategy based on their beliefs regarding the actions of the other players. The goal of this study was to examine, for the first time, the neural correlates of different reasoning levels in tacit coordination games. To that end, we have designed a combined behavioral-electrophysiological study with 3 different conditions, each resembling a different depth reasoning state: (1) resting state, (2) picking, and (3) coordination. By utilizing transfer learning and deep learning, we were able to achieve a precision of almost 100% (99.49%) for the resting-state condition, while for the picking and coordination conditions, the precision was 69.53% and 72.44%, respectively. The application of these findings and related future research options are discussed.

## 1. Introduction

In tacit coordination games, communication between the players is not allowed or not possible. In these games, the more salient solutions, that are often perceived as more prominent [1], are referred to as focal points. While many experiments showed that players are highly successful in converging on the same focal point (e.g., [1,2]), and even though several attempts were made to construct theoretical frameworks to explain this phenomenon (e.g., [3,4,5,6]), there is still no generally accepted explanation of how players manage to converge on the same solution [4]. 

One of the most prominent cognitive models that attempts to explain behavior in tacit coordination games is the level-k model, which is based on the cognitive hierarchy theory [4,7,8]. The model assumes that players’ reasoning depth relies on their subjective level of reasoning, k. For example, players in which k = 0 (sometimes referred to as $L_{0}$ players) will choose randomly between the available actions, while $L_{1}$ players assume that all other players are $L_{0}$ reasoners and will act according to this assumption. That is, $L_{0}$ players might utilize rules but will apply them randomly (picking), whereas $L_{k\geq 1}$ players will apply their strategy based on their beliefs regarding the actions of the other players (coordination). 

In recent years, there has been a growing interest in examining how people reason in strategic situations. However, research regarding the level-k model has been conducted in the context of behavioral game theory, while in this study, we proposed to examine the neural correlates of different reasoning levels in tacit coordination games. To examine different levels of reasoning, we have designed a combined behavioral-electrophysiological study with three different conditions, each resembling a different depth of reasoning: (1) resting state, (2) picking, and (3) coordination. Each participant underwent the three conditions in this sequential order. First, the resting-state EEG was recorded from participants while they were requested to gaze at a cross in the center of the screen. In the second stage of the experiment, participants were engaged in a picking task. Participants were presented sequentially with different sets of four words appearing in Hebrew, and in each of the trials, they were asked to freely pick a word out of each set. In the third stage, participants were presented with the same sets of words that were presented in the picking condition. However, this time participants were engaged in a coordination task. That is, participants were instructed to coordinate their choice of a word with an unknown partner so that they would converge on the same word from the set presented to them in each of the trials. EEG was recorded from participants while they were performing each of the tasks. Before the start of the actual experiment, participants underwent a training session while wearing the EEG cap to get them familiar with the picking and coordination tasks. 

The overarching goal of our study was to classify EEG continuous data into the level-k condition they were associated with. This will show that the level-k model can also be validated by electrophysiological correlates and not only by behavioral indices. This validation may potentially enable the construction of more accurate models for human–agent interactions [9]. To that end, we have first used methods of feature extraction and classification based on conventional machine learning techniques, such as computing the relative energy in each frequency band and applying standard predictive models such as random forest (see Appendix D). These techniques were not proven to be sufficient due to the complexity of the problem and the amount of data such models require. With that in mind, we have used the transfer learning technique (e.g., [10,11,12]) with pre-trained deep learning models trained on large datasets, which we have adjusted to the problem at hand. Specifically, the learning model was based on a continuous wavelet transform 2D image, which has been fed into a pre-trained network (VGG16 trained on ImageNet). Since determining the optimal weights of the different EEG channels is a computationally hard problem, we have defined a relative cost function and optimized the set of weights using a genetic algorithm. Our multi-channel deep learning method achieved a precision of almost 100% (99.49%) for the resting-state condition, while for the picking and coordination conditions, the precision was 69.53% and 72.44%, respectively. 

The contribution of our study is three-fold. First, we have validated the level-k theory in the context of tacit coordination by using electrophysiological data. Second, we have demonstrated that the state-of-the-art transfer learning technique can be useful to cope with a complex classification problem with a low amount of electrophysiological data. Third, we have managed to predict the class label of EEG segments associated with different experimental conditions: picking (level-k = 0), tacit coordination (level-k > 0), or no-task (resting state). The implications of these findings and related future research options are discussed. 

## 2. Materials and Methods

### 2.1. Participants

The participants were 10 students from Ariel University that were enrolled in one of the courses on campus (right-handed, mean age = ~26 years, SD = 4). Each task (picking and coordination) started with a verbal explanation followed by reading a written instruction file. Then, participants signed an informed consent form approved by the IRB of Ariel University. Participants were offered a reward based on the total number of points they earned in both tasks.

### 2.2. Experimental Design

**Figure 1:**
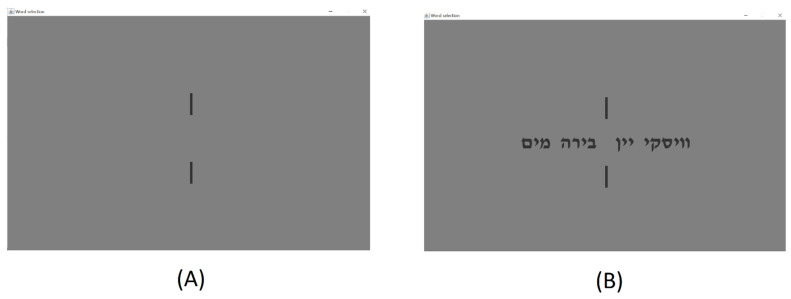
(**A**) Standby screen. (**B**) Game board #1 {“Water”, “Beer”, “Wine”, “Whisky”}.

Experimental conditions comprised resting-state EEG recordings with eyes open for two minutes while participants focused on a red cross on the screen overlayed over a grey background. The next two stages were based on the same set of stimuli and presentation scheme. The experiment consisted of two sets of twelve different trials each with a different set of words. For example, game board #1 displays a trial containing the set (“Water”, “Beer”, “Wine”, “Whisky”) appearing in Hebrew, respectively. Each set of words was displayed between two short vertical lines following a slide containing only the lines without the word set so that participants will focus their gaze at the center of the screen (Figure 1A,B) (e.g., [4,13]). In the first experimental condition, the task presented to the players was a picking task, i.e., participants were only required to freely pick a word out of each set of four words presented to them in each of the 12 trials. Subsequently, participants were presented with the coordination task, comprising the same set of 12 different trials. In the coordination condition, participants were instructed to coordinate their choice of a word with an unknown partner so that they would end up choosing the same word from the set. Participants were further informed that they will receive an amount of 100 points for each selection of a word in the picking task, and for each successful coordination in the coordination task. Each participant sat alone in front of the computer screen during the entire experimental session. It is important to note that no feedback was given between the games. That is, the participants were not informed whether they have coordinated successfully or not with their unknown coplayer. The individual accumulated reward for each of the participants was calculated by randomly matching each participant with a coplayer. The reward was presented to each of the participants only after the completion of the series of games.

**Figure 2:**
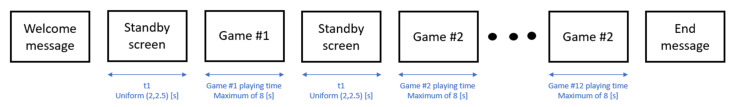
Experimental paradigm with timeline.

Figure 2 portrays the outline of the experiment. Each slide containing the set of words (task trials) was preceded by a slide containing only the vertical lines without the word set (standby slides) to keep the gaze of participants at the middle of the screen throughout the experiment. Each of the standby slides was presented for U(2, 2.5) s, while each slide containing the set of words was presented for a maximal duration of 8 s. Following a task trial, participants could move to the next slide with a button press. The sequence of the task trials was randomized in each session. 

The EEG was recorded from participants while they were performing the tasks. The EEG was recorded by a 16-channel g.USBAMP bio-signal amplifier (g.tec, Austria) at a sampling frequency of 512 Hz, and 16 active electrodes were used for collecting EEG signals from the scalp based on the international 10–20 system. Recording was performed by the OpenVibe (v3.2.0) [14] recording software. Impedance of all electrodes was kept below the threshold of 5 K (ohm) during all recording sessions. 

Before performing the actual experiment, participants underwent a training session while wearing the EEG cap, to get them familiar with the application and task. The training task included a total of five trials (each including a different set of words), as displayed in Appendix C.

### 2.3. EEG Preprocessing and Feature Extraction Using CWT

**Figure 3:**
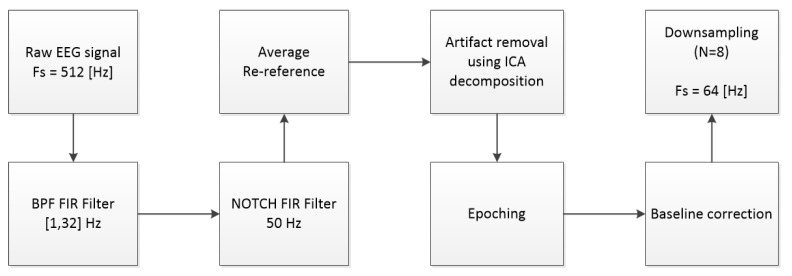
Preprocess pipeline.

Based on the literature (e.g., [15,16,17,18,19]), we focused on the following cluster of frontal and prefrontal electrodes (Fp1, F7, Fp2, F8, F3, and F4). The preprocessing pipeline (see Figure 3) consisted of finite impulse response (FIR), band-pass filtering (BPF) (1,32) Hz, and artifact removal following iCA. The data were re-referenced to the average reference and down-sampled from 512 to 64 Hz following baseline correction. Data were analyzed on a 1 s epoch window from the onset of each game. In the resting-state condition, a 30 to 90 s epoch was extracted from the entire 120 s from trial onset, resulting in 60 1 s epochs per participant. However, in the picking and coordination conditions, there was a total of 12 decision points per participant.

Then, to extract the features on which the processing will be performed, which in this case is an image, we performed the continuous wavelet transform (CWT) calculation. The CWT is a mathematical transformation that gives the signal a complete two-dimensional representation of time and scaling using a wavelet function that receives a continuously changing scale value [20]. We have used the Symlet (e.g., [21,22]) wavelet filter with a scaling factor ranging from 1 to 32. In addition, to obtain optimal results, we examined filters of the order 2, 4, 6, 8, and 10, and the best one according to the cross-entropy (CE) loss function (see Equations (3) and (4)) turned out to be a sixth-order Symlet filter (see Appendix A).

### 2.4. Software Tools and Work Environments

In this study, we used a variety of different tools and environments to perform data collection and analysis. The EEG was recorded by a 16-channel g.USBAMP bio-signal amplifier (g.tec, Austria) using 16 active electrodes based on the international 10–20 system. EEG was recorded by using OpenVibe [14]. The experimental application was developed in Java and included a communication interface based on the TCP-IP protocol to handle triggers.

The pre-processing pipeline (Figure 3) was implemented using EEGLAB [23] (v14.1.1). The CWT and DWT transformations were carried out by the Wavelet Toolbox and Signal Processing Toolbox in MATLAB 2016a. The transfer learning deep models were constructed by using the Keras python package. The optimization process was implemented using the NumPy package in python.

## 3. Results

**Figure 4:**
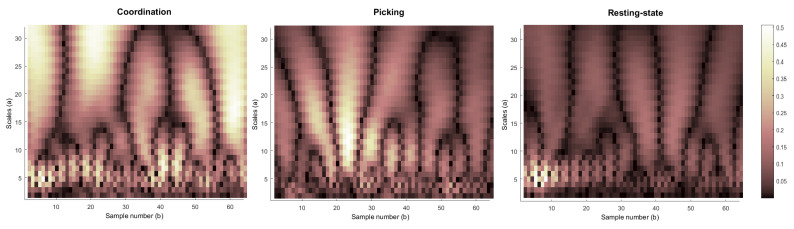
CWT results in different experimental states (resting, picking, and coordination).

**Figure 5:**
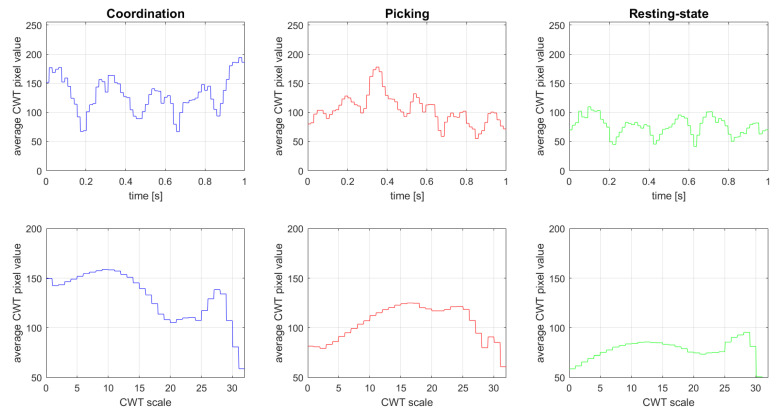
Analysis of Player #3 CWT images as a function of time and CWT scale factor.

Figure 4 shows the CWT conversion results of channel 1 (Fp1) of player #3 in all three experimental states (resting, picking, and coordination). The presented coordination and picking epoch were taken from the same experimental trial containing the same set of words. The x-axis represents the time of the epoch, [0, 1] (s), which is equivalent to 64 samples, while the y-axis represents the CWT value of the corresponding wavelet scaling factor in the range of (1, 32). The wavelet scale corresponds to the frequency of the signal. Note that the higher the scaling factor, the lower the corresponding wavelet frequency. The whole presented analysis can also be seen graphically in Figure 5, which shows the distribution of the average CWT values from Figure 4 according to the time (upper row) and CWT scale (lower row) variables.

Analyzing the differences between the various modes based on the timeline, it can be seen (Figure 4 and Figure 5, upper row) that in the resting-state mode, there is almost no energy increase throughout the epoch. However, in the picking task (level-k = 0), a prominent peak appears after 330 milliseconds (sample 21 out of 64), while in the coordination task (level-k > 0), several prominent peaks, indicating an increase in the signal energy, appear throughout the entire epoch. It can also be observed that the higher frequencies (which are related to smaller CWT scale values) are more dominant in the coordination tasks compared to the picking task and resting-state conditions. This result is consistent with previous studies (e.g., [24,25]) that show that the beta frequency domain (13–30 Hz), especially in the prefrontal brain area, is directly related to brain activity associated with top-down processes associated with prediction and expectation [26].

### 3.1. Cognitive Level Classification Using a Single EEG Channel

**Figure 6:**
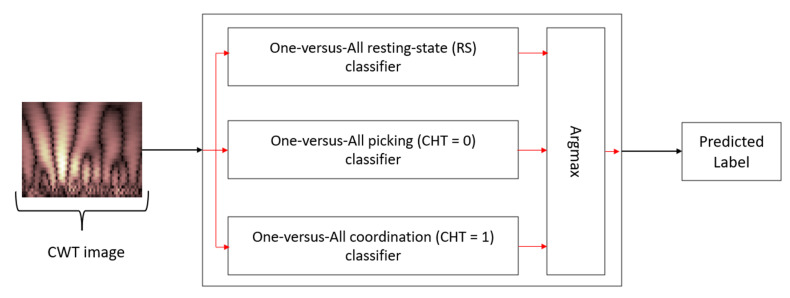
One-versus-all classifier architecture.

To classify the subject’s experimental condition (resting, picking, and coordination) based on the player CWT image during the cognitive task, we have constructed a classification model. However, the small amount of data we have, which includes 840 observations per EEG channel (120 picking epochs, 120 coordination epochs, 600 RS epochs), presents a challenge as it does not allow us to produce a highly complex model. To deal with the problem of the small number of observations in such a complex task, we will take two algorithmic-architectural steps. First, we will implement our classifier according to a one-versus-all approach [27,28]. That is, we will create three different classifiers, each for predicting one of the tree specific labels (resting, picking, and coordination). This approach reduces the complexity of each classifier because its purpose is to identify a single label, which is a more reasonable task given the small number of observations. Consequently, we are now required to train three models instead of one when each model receives a different dataset in which the target images are given the label “1” and all other images the label “0”. The final predicted label will be determined by the highest probability predicted by each of the three models (using an Argmax function). The complete classification architecture for a single electrode based on three one-versus-all classifiers is presented in Figure 6.

Second, we will use a transfer learning method (e.g., [29,30]) that focuses on the use of knowledge generated in a particular problem to solve another problem that has similar characteristics. This enables developing complex models with a low amount of observations [31]. The database used for transfer learning is taken from the ImageNet project [32,33], a visual database used for visual object recognition research that includes more than 14 million tagged images with over 20,000 possible classes.

To produce an optimal prediction model for our problem, classification of the cognitive level (level-k), we will examine three different pre-trained models: InceptionNet V3 [34], ResNet50 [35], and VGG16 [36]. The weights of each pre-trained network were originally calculated to detect one of each of the 1000 different classes (for example zebra, ox, submarine, ambulance, lemon, etc.). The training set on which the networks were trained included about 1.2 million images, while in addition, there were about 50,000 images for validation and about 100,000 images for testing.

**Figure 7:**
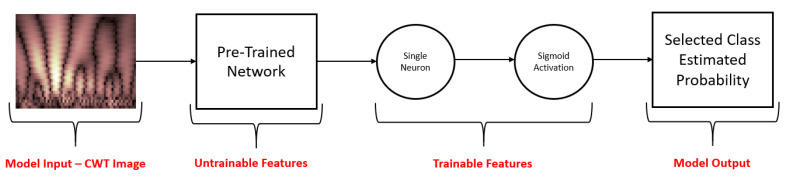
Transfer learning scheme for binary classifier.

In the model training process (Figure 7), we used the abovementioned pre-trained networks to extract the features from the CWT image. The weights of each network were frozen so that they would remain unchanged even at the end of the training process, after which we added a single neuron and a sigmoid activation function after the last pooling layer to obtain a prediction for our problem. The embedding features were taken out of the last pooling layer of the network and not from the output layer because of the difference between the testing set (images of EEG segments) and the transfer learning network training set (ImageNet). When the training set and the testing set comprise images of similar context, it is possible to take the features that are closer to the output layer. However, when the sets are from completely different domains, it is recommended to a take the features from pooling layers residing closer to the middle of the network, which represent basic shapes such as lines, circles, and trends [37]. We preferred using a single-neuron model to more complicated multi-layered architectures due to the size of the training set (for detailed results, see Appendix E). To avoid overfitting, we worked with a four-fold cross-validation method so that the training set included 630 samples at a time (three-fold) and the test set included 210 samples (one-fold). We repeated this process three times to obtain a reliable prediction of all the samples in the test group. The cost function used for the training process was binary cross-entropy (BCE) with balanced weights to overcome the amount of unbalanced observations between the different labels (as there is a 1 to 5 ratio between games and resting-state epochs).

As mentioned in Section 3.1, for each network, we performed five different training sessions. In each training session, we used different sets of CWT images as input. Each set was calculated using a Symlet wavelet of the following different orders: {2, 4, 6, 8, 10}. Therefore, in total, we had 15 configurations for the model (5 wavelets times 3 pre-trained networks). The configuration with the best classification performance for a single channel was obtained by the VGG16 network with a sixth-order Symlet wavelet.

Table 1 presents the classification accuracy results for the different channels. In all channels, the accuracy of the model was higher than the chance level (33.33%). The difference between the best (F3) and worst channels (F7) was 9.65% in the total level of accuracy (81 correctly predicted observations). Finally, there is symmetry between the left side of the scalp and the right side. For each homologous interhemispheric electrode pair (i.e., F7–F8, Fp1–Fp2, F3–F4), the same level of total prediction accuracy can be observed. 

### 3.2. Cognitive Level Classification Using Multiple EEG Channels

In this section, we construct a model which combines the individual electrode models that were presented in the previous section. We define the model as a weighted linear combination of all the different single-electrode models.

(1) $$M\left(a,x\right)=M_{a}\left(x\right)=\frac{\sum_{i\;\in \;S}a_{i}*M_{i}\left(x_{i}\right)}{\sum_{i\;\in \;S}a_{i}}$$

where:

$M_{i}$—The prediction model of the *i*th channel,
$x_{i}$—*i*th channel model input—CWT image of the EEG record from the *i*th channel,
$a_{i}$—The model weight of the *i*th channel.

The result of the weighted model will provide the estimated probability of each of the three conditions, namely, resting state (L = (1;0;0)), picking task (L = (0;1;0)), or a coordination task (L = (0;0;1)). In order to measure the quality of the solution with the various weights, we use the cross-entropy (CE) cost function [38,39] which quantifies the difference between the actual label (i.e., the actual classification value) and the probabilities of the labels predicted by the model:

(2) $$COST\left(M_{a}\left(x\right),L\left(x\right)\right)=\underset{x}{\sum }L\left(x\right)*\log\left(M_{a}\left(x\right)\right)$$

where:

$M_{a}\left(x\right)$—The predicted probability vector by the weighted model for input *x* (which is a CWT image).
L(*x*)—Actual label of input *x* (CWT image).

To obtain an optimal model for the entire dataset of samples in our problem, we calculate the value of the average cost function of all the samples:

(3) $$J\left(a\right)=\frac{1}{m}\overset{m}{\underset{i=1}{\sum }}COST\left(M_{a}\left(x^{\left(i\right)}\right),L(x^{\left(i\right)})\right)=\frac{1}{m}\overset{m}{\underset{i=1}{\sum }}L(x^{\left(i\right)})*\log\left(M_{a}\left(x^{\left(i\right)}\right)\right)$$

where:

m—the number of observations in the dataset.

With that in mind, we seek to find the set of weights that will bring the cost function to a minimum:

(4) $$MIN_{a}\;J\left(a\right)$$

In order to find the set of weights that brings the cost function to a minimum, we have used the genetic algorithm (GA) optimization process (e.g., [40,41]). To evaluate the improvement afforded by the GA model, we have compared it against a baseline model, which was an unweighted model comprising equal weights for each of the six electrodes. GA is an optimization method for hard combinatorial problems that uses a natural selection process, iteratively. In each iteration, the existing set of solutions gradually improves compared to the previous generation of the solution, and this is carried out by biologically inspired operators such as mutation, crossover, and selection.

Specifically, the optimization process started with 50,000 random solutions, where in each iteration, we kept the 15,000 best solutions (using the selection operator). In addition, we have created an additional 20,000 crossover solutions via a pairing of two previous generation solutions selected by the fitness values. Mutation was implemented as random changes in one of the weights in the range between 10% and 50%. In each iteration, we created 15,000 solutions in a mutation configuration, so that in total, we were left with the same amount of 50,000 solutions at the end of each iteration.

Several different and independent runs of the optimization algorithm determined the optimal weights (see Table 2), which resulted in a model with predictive accuracy at the level of 91.66% (770/840) (Table 3), but since our dataset is unbalanced, we will compare the accuracy levels (i.e., true positive rate) of each label, as presented in Table 3. This result represents an overall improved accuracy of 5.09% compared to the best single channel classifier (see Table 1).

Following the optimization process, several insights can be presented. First, the errors of the classifier are only first-order errors. That is, resting-state instances were only mislabeled as picking (level-k = 0) and not as coordination (level-k > 0). By the same token, coordination instances were only mislabeled as picking but not as resting-state instances. Second, the classifier precision (i.e., positive predicted value) is not equal for the different conditions. That is, while for the resting-state epochs the classifier precision is almost 100% (99.49%), for the picking and coordination segments, the precision is 69.53% and 72.44%, respectively. This is probably since the resting-state condition is associated with the spontaneous activity of the brain, whereas the picking and coordination conditions are associated with different levels of task states. It is noteworthy that the imbalance in the amount of data between the different conditions might also contribute to the differences in precision values among the conditions. Similarly, different levels of recall were also observed for the different conditions (98.17% for resting state, 74.16% for picking, and 76.67% for coordination).

**Figure 8:**
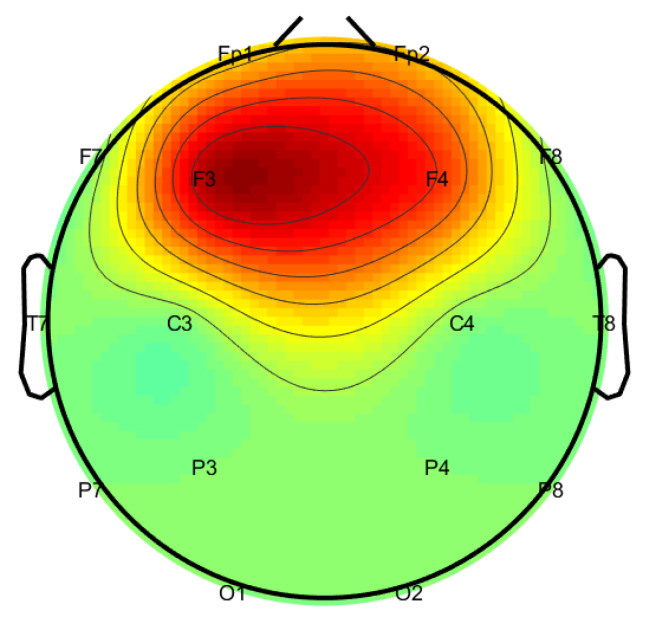
The optimal prediction weights in a 10–20 system.

Figure 8 shows the interpolation of the values of the optimal weights on the scalp according to the 10–20 system. The values of the unused electrodes were set to 0. In this way, it is possible to visually present the relative contribution of each electrode in the combined prediction model.

The level of accuracy of the unweighted model for all classes was lower than the best results of a single electrode. This indicates that the GA model performed better than the baseline model with equal weights.

## 4. Discussion

The overarching goal of our study was to classify EEG continuous data into the level-k condition they were associated with. In this study, we have presented a method to predict the class label of EEG segments taken from three different conditions: two cognitive tasks and a no-task condition (resting state). Each of the two cognitive tasks was associated with a different depth of reasoning, namely, picking (level-k = 0) and tacit coordination (level-k > 0). Classification relied on EEG measures using CWT transformation and transfer learning based on pre-existing state-of-the-art models for object recognition. We have constructed two classification models: the first classification model relied on single-electrode data as input, and the second model optimized the weights of the six frontal and pre-frontal electrodes using a genetic algorithm. 

Additionally, we demonstrated that the state-of-the-art transfer learning technique [10,11,12] can be useful to cope with a complex classification problem with a low amount of data. Specifically, in this study, transfer learning was used to validate the level-k model by classifying electrophysiological data. We have used transfer learning since conventional machine learning models (e.g., random forest, see Appendix D) were not successful in differentiating between picking (level-k = 0) and coordination (level-k > 0). However, the conventional model was successful in differentiating between the no-task condition (resting state) and the two cognitive tasks (picking and coordination). The failure in distinguishing between the two cognitive states could be attributed to the fact the DWT, which is based on 400 ms time windows (with a 50% overlap), was not refined enough to capture the differences between the two conditions. In order to use a higher time resolution, we preferred using CWT. Since this computational method produces an image which requires a large amount of data to distinguish between different geometrical patterns, we opted for using a pre-trained network (VGG16 trained on ImageNet) to embed the CWT image to a feature vector. The feature vectors were used for training an additional neural network which classified between the different conditions.

Transfer learning has been previously used in EEG studies for classification purposes, mainly in the context of clinical research, e.g., [10,11,12]. In these studies, when the problem was relatively simple or in cases where a low amount of data was used, analysis was based on a single-electrode classification [10]. Otherwise, analysis was based on the embedding of multiple electrodes [11,12], to cope with the requirement for a large amount of data. The novelty in our study is that we have trained a separate classifier for each individual electrode and weighted the separate contribution of each electrode for optimal classification results. Training a network with an input layer of six neurons and an output layer of three neurons (resting, picking, and coordinating) with two hidden layers on a small dataset (120 examples of pickers and coordinators each) is not feasible. Therefore, since this study is a multi-class problem with a relatively small and unbalanced amount of data, and given our goal to maximize the accuracy of each classifier, we implemented each classifier based on an input from a single electrode in a one-versus-all architecture. Interestingly, in our study, the topographic distribution of the weights of different models was the strongest over frontal regions (Figure 8). This result is corroborated by previous findings showing a similar distribution, specifically, that cognitive load is enhanced in these regions during on-task periods [42,43]. Overall, the current study presented a novel method for differentiating between cognitive states associated with different depths of reasoning in the context of tacit coordination. Furthermore, to the best of our knowledge, this is the first study to corroborate the level-k theory based on electrophysiological measures.

The findings of our study suggest several avenues for future research. For example, previous studies have shown that various features such as culture [44,45], social value orientation [46,47], strategic profile [48,49], and loss aversion [50,51] might bias decision-making in tacit coordination games. Therefore, it will be interesting to investigate the effect of these parameters on EEG indices. In addition, EEG indices can be used to test assumptions associated with other behavioral economic models, such as team reasoning (e.g., [4,7,52,53]) or cognitive hierarchy theory (e.g., [4,7,8,54]). Furthermore, adding information based on brain sources associated with decision-making in tacit coordination might improve classification accuracy levels. To that end, in future studies, it is recommended to utilize inverse-problem techniques such as LORETA [55,56]. Finally, behavioral and electrophysiological data of human agents (e.g., [2,9,57,58,59]) gained from these studies might aid in constructing brain–computer interfaces as well as autonomous agents. In this study, we used transfer learning when the training set for the embedding network was a general set of images (ImageNet) that did not include EEG signals. It will be interesting to compare the performance of the network when the training set of the embedding network comprises only EEG signals or a mixture of EEG signals and other signal types.

## Figures and Tables

**Table 1:** Classification accuracy as a function of channel number—VGG16 with symlet6 wavelet.

Channel Number (Name)	1 (Fp1)	2 (F7)	5 (Fp2)	6 (F8)	9 (F3)	13 (F4)
Model precision—resting state	94.62% (528/558)	93.15% (517/555)	92.02% (531/577)	94.33% (516/547)	98.62% (571/579)	99.65 (576/578)
Model precision—picking (Level-K = 0)	59% (70/118)	51.63% (63/122)	57.25% (75/131)	53.90% (83/154)	65.89% (85/129)	64.06% (82/128)
Model precision—coordination (Level-K > 0)	56.10% (92/164)	52.15% (85/163)	61.36% (81/132)	56.11% (78/139)	68.18% (90/132)	64.93% (87/134)
Total model accuracy	82.14% (690/840)	79.16% (665/840)	82.14% (690/840)	80.59% (677/840)	88.81% (746/840)	88.69% (745/840)

**Table 2:** The weight values for the different channels in the weighted model after the optimization process.

Channel Notation	(Fp1)	(F7)	(Fp2)	(F8)	(F3)	(F4)
Calculated Weight	0.1216	0.0013	0.1553	0.0108	0.4153	0.2957

**Table 3:** Optimal model using multiple channels—confusion matrix.

		Predicted Classes			True Positive Rate	False Negative Rate
		Resting State (CHT Does Not Exist) L = (1;0;0)	Picking (CHT = 0) L = (0;1;0)	Coordination (CHT > 0) L = (0;0;1)		
**True Classes**	Resting state (CHT does not exist) L = (1;0;0)	589	11	0	98.17%	1.83%
	Picking (CHT = 0) L = (0;1;0)	3	89	28	74.16%	25.84%
	Coordination (CHT > 0) P = (0;0;1)	0	28	92	76.67%	23.33%
Positive Predicted Value		99.49%	69.53%	72.44 %	Total Prediction Accuracy (770/840) 91.66%	
False Discovery Rate		1.51%	30.47%	27.56%		

**Figure A1:**
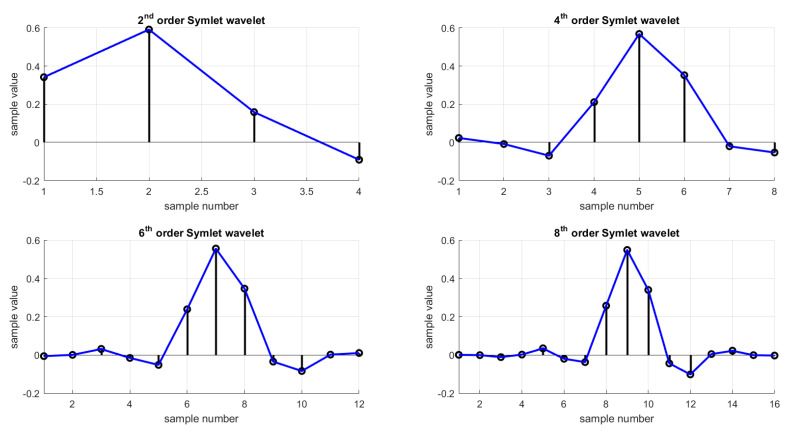
Symlet wavelet function in different orders.

**Figure A2:**
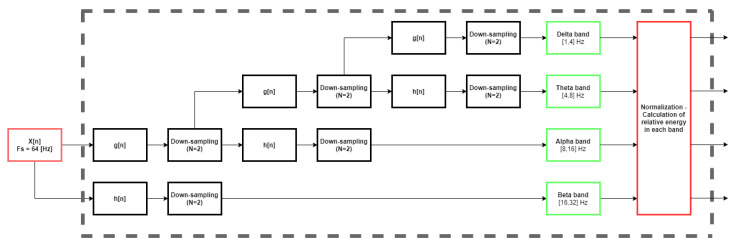
EEG relative band calculation using DWT.

**Figure A3:**
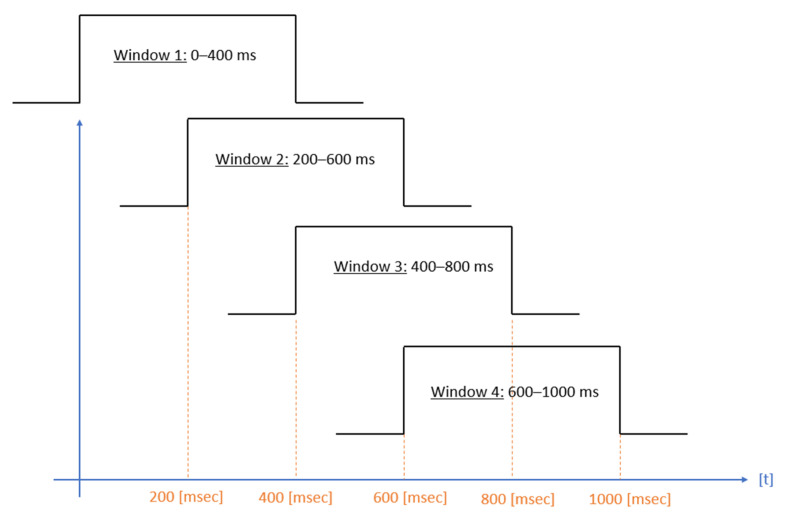
Baseline model windowing scheme.

**Figure A4:**
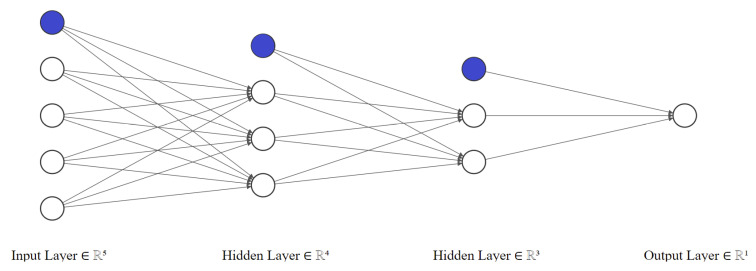
Four-layer trainable neural network model.

## Data Availability

The data presented in this study are available on request from the corresponding author.

## Appendix A. Symlet Wavelet Functions

In this appendix, we will present the Symlet wavelet function in different orders (2, 4, 6, and 8), as can be seen in Figure A1. Figure A1 shows the tradeoff in selecting the wavelet order. While using a higher order wavelet makes it possible to identify more complex patterns, there is a larger number of samples in the wavelet which impairs the temporary resolution of the transduction because now more specimens are involved in each forearm. The problem can also be looked at in reverse, a low order wavelet will provide good time resolution, but its structure is simple, and it will be difficult to find complex patterns in the signal.

In this research, the optimal classified results on a single channel according to the CE cost function (see Equations (3) and (4)) were obtained by a sixth-order wavelet.

## Appendix B. Tacit Coordination Game List

In this appendix, we will describe the set of tacit coordination games, which includes twelve games, that were designed in order to evaluate the individual coordination abilities of the various player together with their electrophysiological patterns in different cognitive hierarchy levels. The full game list is presented in Table A1. It should be noted that the words in the game boards appeared in the Hebrew, which is the native language of the participants.

**Table A1:** Tacit coordination game list.

Game Number	Option 1	Option 2	Option 3	Option 4
1	Water	Beer	Wine	Whisky
2	Tennis	Volleyball	Football	Chess
3	Blue	Gray	Green	Red
4	Iron	Steel	Plastic	Bronze
5	Ford	Ferrari	Jaguar	Porsche
6	1	8	5	16
7	Haifa	Tel-Aviv	Jerusalem	Netanya
8	Spinach	Carrot	Lettuce	Pear
9	London	Paris	Rome	Madrid
10	Hazel	Cashew	Almond	Peanut
11	Strawberry	Melon	Banana	Mango
12	Noodles	Pizza	Hamburger	Sushi

The position of the questions appearing on the game screen as can be seen in Figure 1 is fixed and follows the order of the lists shown in Table A1. This decision in the design of the experiment was made to create a uniform experimental set-up between the various actors and to neutralize the possible effect of spatial cues.

## Appendix C. Training Tasks Game List

This appendix presents the training task, which was performed before the picking and coordination tasks. The purpose of these tasks is to verify the players’ technical understanding of the application before performing the actual experiment. From a review of Table A2, there is no overlap in the content of the training tasks with experiment tasks.

**Table A2:** Training game list.

Game Number	Option 1	Option 2	Option 3	Option 4
1	Sapphire	Glass	Emerald	Diamond
2	Lion	Panther	Frog	Tiger
3	Boat	Helicopter	Bicycle	Plane
4	Thursday	Tuesday	Saturday	Sunday
5	2019	2000	1995	1997

## Appendix D. Classification Using Classical Machine Learning Models

To estimate the performance of a conventional classifier, namely, random forest, we trained a model based on discrete wavelet transform (DWT) [60,61,62], which is the discrete equivalent of the CWT. The DWT was used to calculate the relative energy in each EEG band (Delta, Theta, Alpha, and Beta), as presented in Figure A2.

Four hamming windows with 50% overlap were used. Each window was 400 ms long and contained 26 samples, as can be seen in Figure A3.

Windowing and relative energy computation of each window resulted in 16 features (4 windows with 4 features per window). Based on these features, three classifiers were constructed, one for each class (resting state, picking, coordination) (see Figure 6). Each of the three classifiers was trained using a random forest model with 100 estimators (optimized using grid search) using 4-fold cross-validation. The model was trained on the F3 electrode, which provided the best classification results among all single electrodes.

As can be seen in Table A3, using the DWT and the random forest model, we have managed to differentiate between the no-task state (resting state) and the two cognitive states (picking and coordination), whereas we failed to distinguish between the latter two.

**Table A3:** Baseline model using single channel (F3)—confusion matrix.

		Predicted Classes			True Positive Rate	False Negative Rate
		Resting State (CHT Does Not Exist) L = [1;0;0]	Picking (CHT = 0) L = [0;1;0]	Coordination (CHT > 0) L = [0;0;1]		
**True Classes**	Resting state (CHT does not exist) L = [1;0;0]	520	49	31	86.67%	13.33%
	Picking (CHT = 0) L = [0;1;0]	23	65	32	54.16%	45.84%
	Coordination (CHT > 0) L = [0;0;1]	5	42	73	60.83%	39.17%
Positive Predicted Value		94.89%	41.67 %	53.67 %	Total Prediction Accuracy (658/820) 80.24%	
False Discovery Rate		1.51%	58.33%	46.33%		

## Appendix E. The Effect of Model Complexity on Classification Results

In this appendix we will examine the impact of more complex architectures on the classification results of a single electrode. In addition to the single-neuron architecture, we will examine three additional architectures, with 2, 3, and 4 layers. The structure of the four-layer network can be seen in Figure A4 (bias neurons are painted in blue).

The training process was performed similarly to the process described in the manuscript by using a four-fold cross-validation method. We repeated this process three times to obtain a reliable prediction of all the samples in the test group. In order to fully evaluate the quality of the different models, we will use the F1-score index that weights the precision ($\frac{TP}{TP+FP}$) and recall ($\frac{TP}{TP+FN}$) by a harmonic mean. All the reported results in the appendix are based on using transfer learning with VGG16, that was trained on ImageNet. Since the results obtained for the picking and coordination conditions had similar trends, we present the results for a one-versus-all model that identifies the coordination condition based on the electrophysiological signal. The evaluation of the various models for each of the six frontal and prefrontal electrodes can be seen in Table A4.

**Table A4:** The effect of model complexity on classification results—coordination task (f1 score is the measure by which we evaluate the quality of the model best model colored in green. The least successful model is marked in red).

Electrode/Architecture	(Fp1)	(F7)	(Fp2)	(F8)	(F3)	(F4)
1 layer	precision	precision	precision	precision	precision	precision
	56.10% (92/164)	52.15% (85/163)	61.36% (81/132)	56.11% (78/139)	68.18% (90/132)	64.93% (87/134)
	Recall	Recall	Recall	Recall	Recall	Recall
	76.66%—(92/120)	68.33%—(82/120)	67.50%—(81/120)	65.00%—(78/120)	75.00%—(90/120)	72.50%—(87/120)
	**f1 score = 0.6479**	**f1 score = 0.6007**	**f1 score = 0.6429**	**f1 score = 0.6023**	**f1 score = 0.7143**	**f1 score = 0.6851**
2 layers	precision	precision	precision	precision	precision	precision
	58.17% (89/153)	59.57% (84/141)	59.71% (83/139)	57.66% (79/137)	66.66% (92/138)	64.23% (88/137)
	Recall	Recall	Recall	Recall	Recall	Recall
	74.17%—(89/120)	70.00%—(84/120)	69.17%—(83/120)	65.83%—(79/120)	76.66%—(92/120)	73.33%—(88/120)
	**f1 score = 0.6520**	**f1 score = 0.6437**	**f1 score = 0.6409**	**f1 score = 0.6148**	**f1 score = 0.7131**	**f1 score = 0.6848**
3 layers	precision	precision	precision	precision	precision	precision
	49.67% (75/151)	60.87% (84/138)	59.29% (83/140)	56.30% (76/135)	66.66% (88/132)	63.70% (86/135)
	Recall	Recall	Recall	Recall	Recall	Recall
	62.50%—(75/120)	70.00%—(84/120)	69.17%—(83/120)	63.33%—(76/120)	73.33%—(88/120)	71.66%—(86/120)
	**f1 score = 0.5535**	**f1 score = 0.6512**	**f1 score = 0.6385**	**f1 score = 0.5961**	**f1 score = 0.6984**	**f1 score = 0.6745**
4 layers	precision	precision	precision	precision	precision	precision
	55.88% (76/136)	52.32% (79/151)	57.35% (78/136)	53.64% (81/151)	70.43% (81/115)	63.70%—(79/125)
	Recall	Recall	Recall	Recall	Recall	Recall
	63.33%—(76/120)	65.83%—(79/120)	65.00%—(78/120)	67.50%—(81/120)	67.50%—(81/120)	65.83%—(79/120)
	**f1 score = 0.5938**	**f1 score = 0.5830**	**f1 score = 0.6094**	**f1 score = 0.5978**	**f1 score = 0.6894**	**f1 score = 0.6449**

Table A4 displays precision and recall scores for each combination of electrodes and number of layers, as well as the F1 score (the weighted average of precision and recall) for each combination of electrodes and number of layers. The table shows that more complicated models with more layers do not improve the F1 score compared to the simplest single-neuron model. This finding is probably due to overfitting created by complicated models applied on a relatively small amount of information. Consequently, we decided to stay with the simplest single-neuron model, which is the most parsimonious one.
